# Near-Field Direct Write Electrospinning of PET-Carbon Quantum Dot Solutions

**DOI:** 10.3390/ma17246242

**Published:** 2024-12-20

**Authors:** Fatemeh Mohtaram, Michael Petersen, Maria Ahrenst-Mortensen, Liva Skou Boysen, Frederik Hejgaard Gram, Helene Halsen Malling, Noah Frederik Hallundbæk Bang, Yan Jurg Hess, Peter Fojan

**Affiliations:** Materials Science and Engineering Group, Department of Materials and Production, Aalborg University, 9220 Aalborg, Denmark; fatemehm@mp.aau.dk (F.M.); mpete23@student.aau.dk (M.P.); lboyse23@student.aau.dk (L.S.B.); fgram19@student.aau.dk (F.H.G.); hmalli23@student.aau.dk (H.H.M.); nbang23@student.aau.dk (N.F.H.B.); yhess22@student.aau.dk (Y.J.H.)

**Keywords:** near field direct write electrospinning, carbon dots, PET solution electrospinning, fluorescence labeled PET nanofibers, cellulose electrospinning

## Abstract

Electrospinning of polymer material has gained a lot of interest in the past decades. Various methods of electrospinning have been applied for different applications, from needle electrospinning to needleless electrospinning. A relatively new variation of electrospinning, namely near-field electrospinning, has been used to generate well-defined patterns. This variation of electrospinning, also known as near-field direct-write electrospinning, allows for precise control of the fiber deposition, sacrificing on the thickness of the resulting fibers. Typically, for this method, melt electrospinning is preferred, since it provides a higher viscosity of the polymer and thereby better control of the fiber deposition. However, when mixing additives into the spinning dope, a solution spinning approach is preferable since it provides a more homogeneous distribution of the additives in the spinning dope. A fluorescent spinning dope of dissolved PET with fluorescent carbon quantum dots has been used to generate the fluorescent patterns. These can be used to generate logos, bar codes, or QR codes to encode information about the material, such as watermarks or counterfeiting tags.

## 1. Introduction

Electrospun nanofiber technology has garnered widespread interest for its adaptability, enabling the creation of intricate designs and multifunctional structures from a broad spectrum of materials and innovative fabrication approaches. These features give them unique properties that can be applied in various applications such as food packaging [1], membranes for wastewater treatment [2], ultra-flexible batteries [3], sensors [4], and solar cells [5]. The electrospinning process requires a high-voltage power supply (HVPS), a syringe with a steel needle or capillary tube, and a metal collector. When an electrically conductive precursor solution is subjected to a critical electric field, surface tension is overcome, forming a Taylor cone. The cone’s tip ejects a charged jet, initiating the electrospinning process [6]. As the jet travels through the air, evaporation and charge dissipation cause it to thin and destabilize during the jet thinning phase. It eventually solidifies and deposits as fibers onto the collection surface in the final solidification step. The needle-to-collector distance directly affects the electric field strength, influencing the spinning jet’s trajectory, deposition time, and fiber morphology [7]. When the spinning head operates in a translational 3D space, solution electrospinning transitions to near-field electrospinning (NFE). NFE is categorized as additive manufacturing technologies, commonly referred to as 3D printing, and occurs when the nozzle-to-collector distance is reduced to a few millimeters or less, minimizing jet bending and whipping [8]. Apart from the reduced distance between the needle and the collector, the system setup remains identical to standard electrospinning. Reducing the distance to 500 μm–3 mm helps prevent jet instability, such as bending or whipping. This shorter distance ensures the use of the stable region of the electrospun jet near the needle, making the system particularly suitable for direct writing applications [9]. This produces a straight jet perpendicular to the collector, enabling resolutions of several hundred nanometers in printed constructs [8,10]. Sun and colleagues were the first to introduce the near-field electrospinning process and coined the term “NFES”. Their main goal was to fabricate aligned, continuous, and controllable fibers ranging from 50 to 500 nm, addressing the limitations of traditionally electrospun random fibers. The random deposition of fibers often restricts scaffold design possibilities; therefore, techniques such as NFES and melt electro-writing (MEW) were developed to provide solutions for achieving orderly fiber deposition [11,12]. Near-field electrospinning is an advanced technique for producing micro- to nano-scale fibers with precise alignment and control. It can be used to create microfibers with large pores and, when combined with solution electrospinning, can fabricate substrates with optimal pore sizes and porosity [13]. In 2003, Kameoka et al. introduced a method to control individual electrospun fibers by minimizing the air gap distance and moving the polymer spinneret relative to the collector to prevent bending instabilities with precise movement between the polymer source and the collector; this technique enables the direct “writing” of fibers, allowing for the creation of specific geometries in a controlled, layer-by-layer manner [11]. Wang et al. utilized NFES technology to deposit a poly(vinylidene fluoride-co-trifluoroethylene) (P(VDF-TrFE)) nanofiber mesh with an average fiber diameter of approximately 370 nm. These nanofibers were used as a broad-bandwidth acoustic sensor based on a piezoelectric nanofiber mesh [14]. Poly(vinylidene fluoride) (PVDF) microfiber sensors for detecting weak mechanical excitations were produced using the NFES technique. PVDF microfibers were electrospun onto a Cu/PET substrate with a distance between the needle and collector of 1.5 mm. Due to in situ polarization during the NFES process, the PVDF microfibers exhibit piezoelectric properties, enabling them to function as self-powered sensors and nanogenerators for sensing and harvesting mechanical energy [15]. George et al. achieved control over the straightness, diameter, and inter-wire spacing of graphitized carbon nanowires. The carbon nanowires were fabricated by stabilizing patterned polyacrylonitrile (PAN) wires, which were obtained using near-field electrospinning, followed by pyrolysis [16]. A recent study introduced an innovative approach to developing a 2D circular-shaped polyvinylidene fluoride (PVDF) fiber piezoelectric sensor using a direct-write near-field electrospinning system. Key parameters such as voltage, feeding rate, and needle-to-collector distance were optimized using the uniform design method. The sensor’s 2D circular fibers, integrated with radial electrodes and polyethylene terephthalate (PET) film, were shown to exhibit effective multi-directional sensing capabilities, demonstrating its potential in dynamic behavior measurements [17]. A recent study by Huang et al. introduced a highly sensitive and anisotropic strain sensor fabricated using near-field electrospinning. The resulting strain sensor exhibited a high gauge factor and excellent stretchability, making it ideal for applications in robotic sensing and health monitoring [18]. The development of flexible piezoresistive (PR) sensors using near-field electrospinning was conducted by Huang et al. by introducing a grid-like polyurethane fiber spacer layer at the interface, which improved sensor characteristics. This strategy provides a universal method to improve the performance of contact-dominant sensors without altering the active materials, making it a promising technique for flexible electronics and wearable sensors [19]. This research demonstrates the significant potential of near-field electrospinning for precise fabrication of polymeric membranes with controlled pore structures, making it a promising technique for applications in filtration, tissue engineering, and advanced material development.

PET represents the single largest volume of fibers consumed worldwide, making it the most extensively used synthetic fiber. It is a semicrystalline thermoplastic polyester produced through the esterification of terephthalic acid and ethylene glycol [20,21]. Polyethylene terephthalate nanofibers, commonly known as polyester fibers, are widely used in the biomedical field [22], packaging [23], and filtration [24]. Recently, carbon quantum dots (CQDs) have gained significant attention as novel luminescent indicators. These emerging carbon nanomaterials, typically less than 10 nm in size, possess several unique properties, including low toxicity, eco-friendliness, extremely low density, and excellent optical characteristics. Due to these features, CQDs have found wide applications in fields such as drug delivery, catalysis, sensing, bioimaging, and solar cells [25,26,27]. Given the limited literature on near-field electrospinning, we aimed to contribute to this emerging field for the first time, to the best of our knowledge. By exploring new methodologies for producing PET nanofibers through NFES, we sought to expand the understanding and potential applications of this technique. On the other hand, carbon quantum dot nanoparticles were utilized as fluorescent agents to produce fluorescent composite nanofibers. The impact of these fluorescent agents on the optical properties of the nanofibers was evaluated by incorporating CQD nanoparticles at various concentrations. By successfully depositing uniform PET fibers and incorporating fluorescent carbon quantum dots, we were able to create logos and scannable QR codes using near-field electrospinning technology. Different concentrations of PET solutions, both with and without CQDs, were spun using a 3D printer, and the electrospun PET mats were covered on both sides with a cellulose acetate NF layer.

## 2. Material and Methods

### 2.1. Materials

Cellulose Acetate (CA), $M_{N}$ 30,000 GPC was supplied from ALDRICH Chemistry. Sodium hydroxide (NaOH) and dichloromethane (DCM) were purchased from VWR LIFE SCIENCE. Trifluoroacetic acid (TFA) was supplied from Iris Biotech and polyethylene terephthalate powder (PET) from GoodFellow. Ethanol was purchased from Avantor (Radnor Township, PA, USA) and acetone from Sigma Aldrich (St. Louis, MO, USA). All materials were used without further purification.

### 2.2. Preparation of Spinning Dope

Polyethylene terephthalate powder solutions were prepared using trifluoroacetic acid as the solvent [28]. The PET concentration in the solutions ranged from 25% to 40% (*w*/*v*). The optimal concentration was found to be 35% (*w*/*v*). The solutions were stirred overnight at room temperature to ensure homogeneity. The resulting polymer solutions were doped with 0.05% CQDs and mixed thoroughly until uniform. Additionally, other CQD concentrations, ranging from 0.05% to 0.2%, were also investigated.

To increase the volatility of the spinning dope, dichloromethane was introduced as a co-solvent. Both 30:70 and 50:50 (*v*/*v*) DCM/TFA solutions were tested, with PET and CQD concentrations in the aforementioned ranges. The solutions were stirred overnight before use to ensure homogeneity.

For the generation of nonwoven fiber mats with far-field electrospinning, solutions of cellulose acetate powder and acetone as solvent were prepared [29]. The CA concentration was kept at 12.5% (*w*/*v*) for all experiments. The solutions were stirred until homogeneous before use.

### 2.3. Electrospinning Process

**Figure 1:**
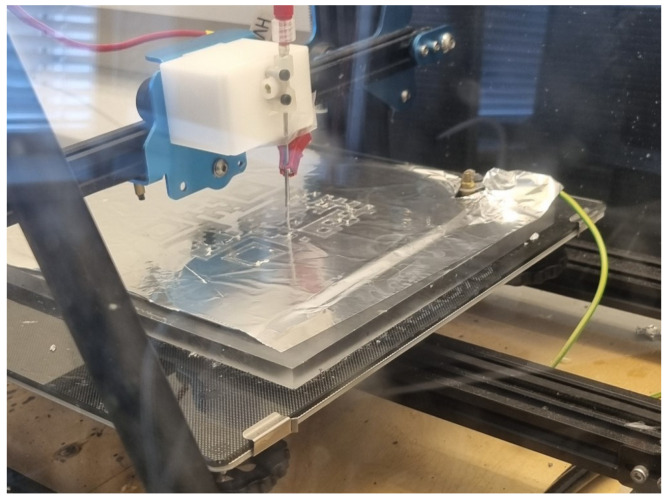
The near-field direct-write electrospinning setup writing a QR code from a 40% PET/TFA solution.

The near-field electrospinning setup consisted of a Creality CR-10S Pro V2 3D printer as the spinning platform [30]. The melt printing head on the Z/X axis of the 3D printer was replaced with a needle, connected to a syringe pump transporting the spinning dope from a glass syringe to the tip of the needle. The near-field spinning setup is shown in Figure 1.

A 3 kV high-voltage power supply was connected to the needle with a crocodile mount, and the collector plate was connected to ground. Each sample has been created on a piece of aluminum foil placed on top of an insulating layer of glass separating the collector and printer bed to protect the control electronics and Nema 17 motors of the 3D printer. For the same reason, the needle has been mounted on a block of Teflon, as can be seen in Figure 1.

After the syringe pump was started and a droplet had formed at the end of the needle, the high-voltage supply was turned on and the spinning process started. Once stable electrospinning has been achieved, the programmed pattern of the G-code was initiated. To establish the electrospinning parameters for solution direct writing with different spinning dopes, a simple grid pattern was used for testing. The grid patterns were created with the program Ultimate Cura 5.7.0 [31], which exports the pattern as G-code. Other patterns, such as a QR code were generated with the help of an online QR code generator [32]. The logo associated with Aalborg University, as well as the QR code, was converted into an STL file, which can be read by Ultimate Cura and translated into a printable G-code with the corresponding printing parameters of the Creality CR-10S Pro V2 3D printer. When printing the QR code, the program was set to move the printer head 0.5 mm up after the first layer to accommodate for the shortened distance from the created fibers. Afterward, four additional layers were printed at the new height.

To release the logo from the aluminum, it was placed face down on a piece of paper in a petri dish and was etched with 2 molar NaOH for 10 min. By carefully removing the aluminum foil, the logo is then laid on the piece of paper, making removal straightforward. Furthermore, the logo was placed between two new pieces of paper and rinsed with water through the paper to preserve the structure. It was then completely dried in a heating cabinet before being stacked upon a nonwoven CA mat. To conceal the watermark pattern created with near-field direct write electrospinning, the logo was embedded within far-field electrospun layers of CA.

### 2.4. Preparation and Characterization of Carbon Quantum Dots

Carbon quantum dots were prepared via pyrolysis following reported methods of carbon synthesis using citric acid and urea as precursors [33,34]. Pyrolysis was achieved by heating a solution adjusted to a mass ratio of 2:1 urea to citric acid in a common microwave at 650 W for four minutes. Post-pyrolysis, the CQDs were suspended in Milli-Q water and sonicated for one hour. The suspension was then centrifuged at 3000 rpm for 20 min, and only the supernatant was retained. Ultraviolet/visible (UV/Vis) spectroscopy was performed to record the light absorbance of the carbon quantum dots using a Shimadzu UV-1800 spectrophotometer in the range of 300 nm to 600 nm. The wavelength of maximal absorbance was used as the excitation wavelength for fluorescence spectroscopy, which was conducted using an ISS Chronos DFD fluorescence lifetime spectrometer coupled to an ISS K520 digital frequency domain and a P110 lamp power supply. Emission measurements were made by scanning in 1 nm steps with an accumulation time of 0.3 s. Measurements were made on diluted samples from the stock solution. Dynamic light scattering (DLS) measurements were performed using a Brookhaven NanoBrook Series instrument, and measurements were analyzed with the BIC Particle Solutions software. The measurements were taken at 90 ° angle, at 25 °C, for a total of 30 s per measurement and 30 s equilibration time. The expected particle size range of 10 nm to 50 nm was set in the measurement window. Ten automated measurements were taken, and a refractive index of 1.693 was used for the sample parameters [35]. The carbon quantum dot stock was lyophilized for long-term storage.

## 3. Results and Discussion

### 3.1. Carbon Quantum Dots Synthesis and Characterization

**Figure 2:**
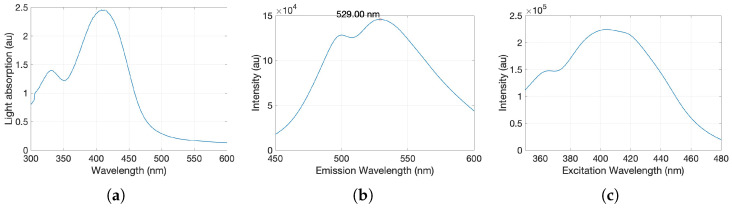
Photoluminescence characterization of the produced carbon quantum dots. (**a**) UV/spectroscopy results depicting the intensity of light absorbance (au) with respect to wavelength. (**b**) fluorescence spectrometry emission spectrum for 410 nm excitation wavelength, showing emission maxima at 529 nm. (**c**) fluorescence spectrometry excitation spectrum for emission of 529 nm wavelength.

Carbon quantum dots were successfully synthesized with the above-mentioned method. The carbon quantum dots showed strong fluorescence in the visible wavelength regime when excited with a 314 nm UV lamp. This is also in agreement with the reported fluorescence of similarly produced CQDs [33,34]. Absorption spectroscopy of the CQDs showed a global maximum at 410 nm (Figure 2a), which has been used as the excitation wavelength for fluorescence emission measurements (Figure 2b). In addition, the particle’s excitation spectrum was measured to assess the ensemble of wavelengths contributing to the observed fluorescence emission (Figure 2c).

**Figure 3:**
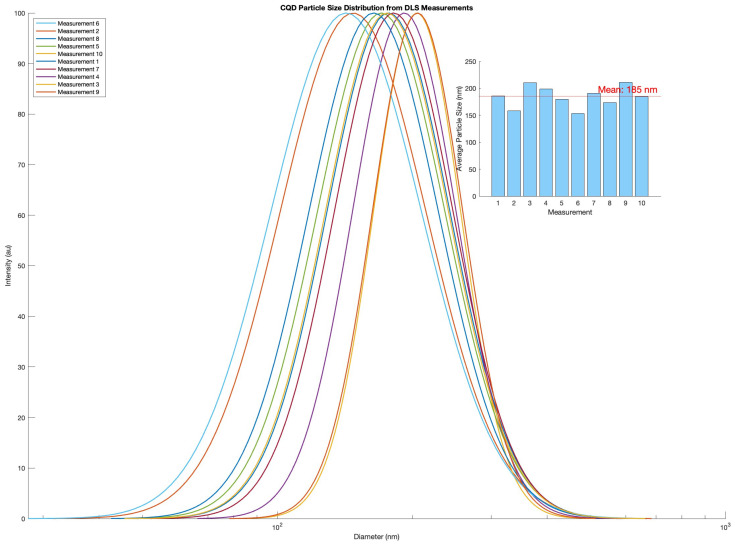
DLS distribution of CQD measurements conducted at a second time, x-axis in logarithmic scale. Inset: Histogram of the mean diameter estimations from DLS measurement with a red line indicating the average mean diameter estimation at 185 nm.

The calculated diameter from the DLS experiments based on five consecutive measurements yielded a broad size distribution around 21 ± 11 nm, which is in good agreement with the findings of Qu Songnan et al. [34] using transmission electron microscopy and atomic force microscopy. DLS measurements of CQD diameter measured after some storage time yielded consistently larger diameters than the first measurements of freshly prepared CQDs. The average diameter of the aged sample of CQD was calculated to be 185 nm (Figure 3). This value is based on ten measurements with two replicas. The particle’s photoluminescent properties were consequentially re-inspected after the difference in size estimations was measured, yet they presented the same characteristics as previously found. Increases in size estimations of CQDs using DLS have been reported to be associated with long-term degradation of fluorescent impurities depositing on CQDs over time [36] and were neglected in light of the consistent photoluminescent profile.

### 3.2. Near-Field Direct Writing of PET Spinning Dope

The experiments consisted of three segments: a simple grid pattern to evaluate viable parameters, a logo to test the ability to create specific shapes, and embedding them within nonwoven, far-field, electrospun mats where the shapes become fluorescent under UV light due to added CQDs. Lastly, a QR code was created to test the precision of the setup and explore the possibility of preserving data in electrospun fibers.

For the grid pattern, it was observed that the distance between the collector and the needle gave the most reliable results, around 5 mm, with a voltage of around 2.5 kV. The flow rate was in the range of 0.6 to 0.8 mL/h. Difficulties occurred when attempting to deposit fiber on top of the fibers, due to either the precision of the 3D printer of ±0.1 mm [30] or electrostatic charge repulsion between wet deposited fibers and incoming fibers. These parameters are within the typical ranges of near-field electrospinning [12].

Electrospinning a spinning dope with only TFA as solvent produced almost exclusively fibers that merge at the intersections due to excess solvent. To reduce this effect, DCM was added as a co-solvent to increase volatility [37]. As the humidity was not controllable in this setup, the ratio between solvent and co-solvent was adjusted to accommodate changes in humidity. It was observed that a sample may contain both wet and dry fibers. An example of this can be seen in Figure 6a, where both thick, nonuniform fibers and thin, uniform fibers are present. The humidity has a large impact on the electrospinning process, which can lead to inconsistent fiber diameters [38].

The speed of the 3D printer was fixed at 200 mm/s. The relative speed between the collector and jet was therefore adjusted through the processing parameters for a solution or by adapting the spinning dope. It has previously been shown that an increase in the relative speed between jet and collector results in thinner fibers [39] and reduces fiber curling [40]. In addition, Hutmacher et al. [41] have previously shown in melt electrospinning that the collector speed needs to match the jet speed to obtain straight fiber deposition and that a change in the jet speed, such as lowering the flow rate, has to be accommodated by a corresponding change in collector speed.

**Figure 4:**
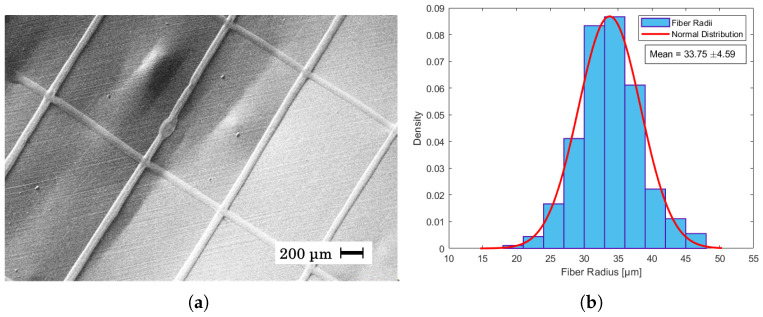
(**a**) SEM image of fibers created from a spinning dope of 35% PET in 30:70 DCM/TFA with 0.1% CQDs, a distance of 5 mm, a voltage of 2.43 kV, and a flow rate of 0.8 mL/h. The temperature was 20.5 °C, and the relative humidity was 40.1%. (**b**) Fiber radii distribution of SEM image measured in μm. The average fiber radius was measured to be 33.75 ± 4.59 μm.

**Figure 5:**
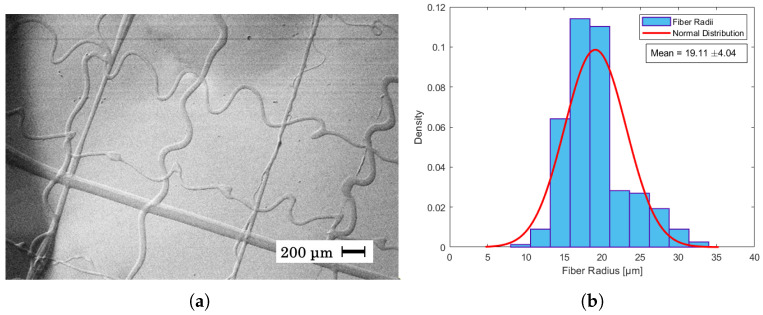
(**a**) SEM image of fibers created from a spinning dope of 35% PET in 30:70 DCM/TFA, a distance of 4 mm, a voltage of 2.55 kV, and a flow rate of 0.6 mL/h. The temperature was 21.5 °C, and the relative humidity was 34%. (**b**) Fiber radii distribution of SEM image measured in μm. The average fiber radius was measured to be 19.11 μm ± 4.04.

**Figure 6:**
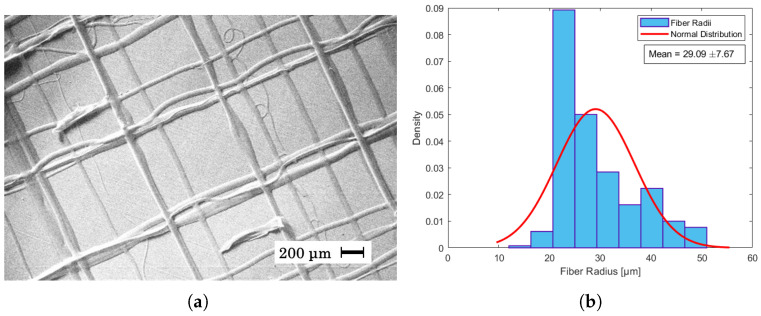
(**a**) SEM image of fibers created from a spinning dope of 35% PET in 30:70 DCM/TFA with 0.1% CQDs, a distance of 5 mm, a voltage of 2.43 kV, and a flow rate of 0.8 mL/h. The temperature was 20 °C, and the relative humidity was 41.1%. (**b**) Fiber radii distribution of SEM image measured in μm. The average fiber radius was measured to be 29.09 μm ± 7.67.

In Figure 4a it can be seen that the given parameters resulted in straight fibers with an average fiber radius of 34 μm ± 4.59 as shown in the histogram of Figure 4b. This is in contrast with Figure 5a, where a higher flow rate and humidity led to a higher jet speed, and as the collector speed was held constant, wavy fibers appeared. In near-field electrospinning, a shorter distance between the needle and the collector can result in wavy fibers due to the rapid deposition of fibers before they have a chance to fully solidify. This is often caused by the incomplete elongation of the polymer jet at short deposition distances, leading to irregular fiber alignment or wavy morphologies [40,42]. The average radius of these fibers was measured to be 19 μm ± 4.04 as seen in Figure 5b. Despite being able to create straight fibers in Figure 6a, there is a general shift in the grid from layer to layer, which is assumed to be mechanical as the shift becomes consistently larger from left to right. The fibers of the shifted grid were measured to be 29 μm ± 7.67 (Figure 6b). This is the first time that PET has been used in near-field direct-write electrospinning from solution. Although other polymers have been used in near-field direct-write electrospinning and also yield thinner fibers, none of those have been achieved from solution [43,44].

### 3.3. Solution Direct Write Electrospinning Containing Carbon Quantum Dots

**Figure 7:**
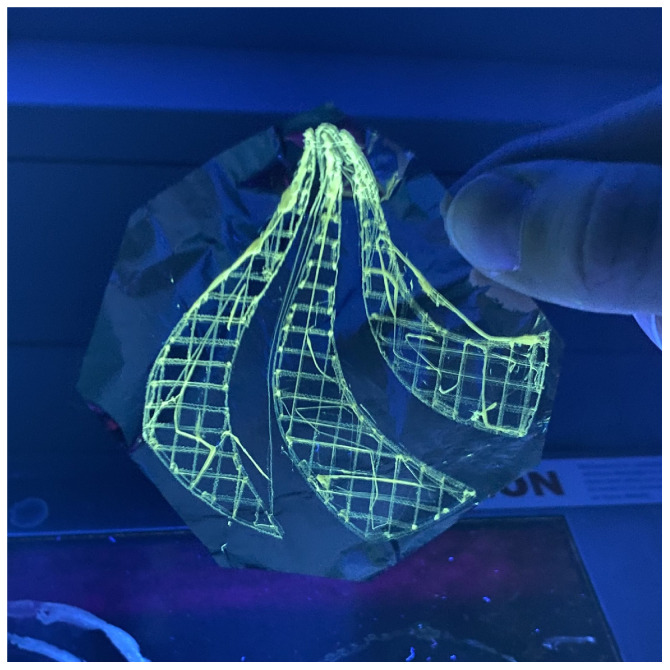
A fluorescent AAU logo on aluminum foil, in UV light. Fibers created from a spinning dope of 40% PET in 30:70 DCM/TFA with 0.2% cQDs, electrospun at a distance of 4 mm, a voltage of 2.6 kV, and a flow rate of 0.8 mL/h at room temperature and a relative humidity of 36%.

A PET solution of 40 wt% in 30:70 DCM/TFA with 0.2% CQDs has been deposited as thin fibers with direct-write near-field electrospinning in the form of a logo seen in Figure 7. Although the fiber placement is not perfect over the whole structure, the logo itself is well resolved and recognizable. The logo had to be structurally stable enough to be removed from the underlying collector foil and transferred onto the CA matrix.

**Figure 8:**
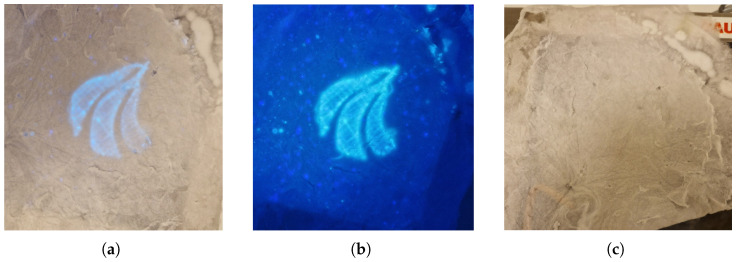
AAU logo was spun (**a**): Under UV light, in ambient light. (**b**): Only UV light, and without ambient light, and (**c**): Only ambient light, and no UV light. With 30% PET in 30:70 DCM/TFA and 0.05% CQDs, spinning dope, made in near-field encased in nonwoven CA mat of 12.5% CA in acetone spinning dope, made in far-field.

The CQDs cause a yellow tint, visible in ambient light, and add fluorescent properties to the spun fibers, which is more apparent at high concentrations (above 0.1 wt% CQDs in solution). Imperfections in the CA matrix originate from residuals being released from the logo when not properly rinsed, revealing the logo due to excess NaOH in the fibers. The resulting mat had a texture similar to cotton or wool, as seen in the top right corner of Figure 8a. The logo was visible through the CA mat in ambient light when the concentration of CQDs in the spinning dope was above 0.05%. In Figure 8, the logo was spun from a spinning dope containing a concentration of 0.05% CQDs, and the logo is still visible under UV light but concealed when lit only by ambient light.

The main objective was to create a recognizable logo outline and fill it in. The fibers within did not have to be dry or uniform, nor lay exactly on top of each other, as scaffolding was not the goal. However, some precision is required to obtain a proper resolution. The importance of these factors increases as the size of the figure decreases and is even more paramount in the case of a QR code that has to be scannable.

### 3.4. Precision and Information Storage with Solution Near-Field Direct Write Electrospinning

**Figure 9:**
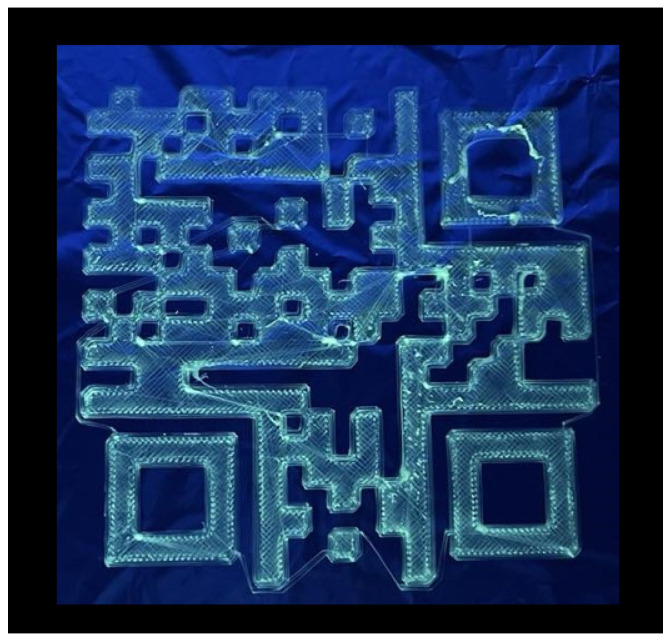
QR code created from a spinning dope of 28% PET and 0.05% CQDs in TFA, a spinning distance of 2 mm to 2.5 mm, voltage of 1.5 kV, flow rate of 0.55 mL/h and needle diameter of 2 mm. The temperature was 21.5 °C and the relative humidity was 47%. This QR code is approximately 150 × 150 mm in size.

A QR code was created to examine the precision of the fiber deposition. The QR code, representing the letters “AAU”, was chosen and written using the same method as the grid and logo. To allow for better resolution of the QR code, the amount of small isolated squares was limited. Since the solution near-field direct write electrospinning is continuous, thin fibers were deposited in between individual shapes, forming bridges between each section, as can be seen in Figure 9. The thicker fibers dragged along the surface of the QR code seen in Figure 9 are the result of partial drying of the Taylor cone, which is dragged from the needle onto the surface. These fibers did not have any effect on the readability of the QR code, since the error margin of a QR code can be up to 30% of its entire area before it is no longer readable [45]. As can be seen in Figure 9. The thicker fibers being formed due to partial drying of the Taylor cone are only of concern if they are placed outside the QR code structures.

Increasing the size of the QR code leaves more room for error at the expense of the possible applications and production time. The QR code presented in Figure 9 was machine-readable when illuminated with UV light. In order to increase the precision of the solution, direct-write electrospinning and miniaturization of the QR code down to the mm or even sub-millimeter range, the jet would have to be stopped to avoid bridging. Enabling an easier transfer of the QR code, a direct spinning onto the nonwoven mat or fabric, and subsequently covering it with a top layer would be preferable for production purposes. Alternatively, a support mat of fibers without the CQDs could be written underneath the QR code to keep everything in place and transfer the whole patch onto the fabric.

The spinning dope with DCM as a co-solvent for the QR code turned out to dry too fast and resulted in the deposition of dried patches of PET/CQDs onto the surface, rendering the QR code pattern unreadable. A spinning dope consisting of TFA as the sole solvent resulted in the successful creation of a machine-readable QR code. During the near-field direct write solution electrospinning, in contrast to classical far-field electrospinning, a relatively high humidity turned out to be optimal since an increase in precision was observed due to the lower evaporation speed of the solvent. However, this is a fine balance since excessive humidity results also in wet fibers that start to merge and melt into each other, forming a polymer film instead of polymer fibers in the desired shape. A polymer film embedded into a fabric will always be perceived as a disturbance in the fabric, and therefore isolated fibers are preferred.

The applications and possible alterations of the solution near-field direct write electrospinning are vast. The main advantage of electrospinning homogeneous spinning dopes is the creation of new composite materials, such as fibers with CQDs. Near-field direct-write electrospinning from solution allows for the generation of mixed polymer fibers and composite material (e.g., fiber-CQD composite).

## 4. Conclusions

By using solution near-field direct-write electrospinning, the deposition of dry, uniform, aligned fibers was demonstrated. The method allows for the writing of any possible pattern that could be shown with the creation of a logo and a machine-readable QR code. These patterns can be integrated seamlessly into other fabrics, as demonstrated for a nonwoven CA mat with the logo embedded into it and only being visible under UV light. This method holds great promise for various applications ranging from watermarking to material labeling for circular economy and recycling.

## Data Availability

The original contributions presented in this study are included in the article. Further inquiries can be directed to the corresponding author.
